# MODIFIABLE RISK FACTORS FOR ALZHEIMER'S DISEASE: CURRENT STATUS AND OPPORTUNITIES FOR ACTION

**DOI:** 10.1093/geroni/igac059.511

**Published:** 2022-12-20

**Authors:** John Omura

**Affiliations:** CDC, Atlanta, Georgia, United States

## Abstract

In 2021, the National Plan to Address Alzheimer's Disease included a new goal to address risk factors for ADRD. The Behavioral Risk Factor Surveillance System (BRFSS) assesses several modifiable risk factors in its core survey and subjective cognitive decline (SCD), which may be an early indicator of developing ADRD, in the optional Cognitive Decline module. To assess the current status of modifiable risk factors in the US and identify opportunities for public health action, data from the 2019 BRFSS were examined. Prevalence of eight modifiable risk factors for ADRD and proportion of respondents with total number of risk factors was estimated among respondents aged ≥45 years, overall, by SCD status, and by selected demographic characteristics. Findings can inform strategies and priorities to support the National Plan’s new goal to reduce modifiable risk factors to help delay onset or slow progression of ADRD and its symptoms.

